# A comparative study of alkaloid and phenolic compounds in different organs and tissues of *Berberis integerrima*

**DOI:** 10.1371/journal.pone.0321255

**Published:** 2025-05-19

**Authors:** Nastaran Zhaleh, Mohsen Sharifi, Elaheh Samari, Mohammad Reza Halvagar, Seyedeh Hanieh Zeidi

**Affiliations:** 1 Department of Plant Biology, Faculty of Biological Sciences, Tarbiat Modares University, Tehran, Iran; 2 Department of Inorganic Chemistry, Chemistry and Chemical Engineering Research Center of Iran, Tehran, Iran; Institute for Biological Research, University of Belgrade, SERBIA

## Abstract

*Berberis integerrima* Bunge is a valuable plant of the Berberidaceae family used in medicine and industry. The properties of the different parts of this plant vary and depend on the distribution of its secondary metabolites, including alkaloids and phenolic compounds. Here, we aimed to evaluate and compare phenolics and alkaloids of the different organs and tissues of *B. integerrima*. Our results showed that the highest content of phenolic compounds was found in the fruit and leaf, while the total alkaloid level was higher in the root. In the fruit, leaf and stem, the main phenolic acids were caffeic acid, cinnamic acid and gallic acid, respectively. In contrast, the highest levels of ferulic acid, catechin, resveratrol and luteolin were detected in the root. The highest content of berberine, one of the most important alkaloids of barberry, was found in the root, especially in the bark tissue. Further experiments showed that phenolic compounds and berberine, in the aerial organs and root of *B. integerrima*, respectively, are likely responsible for the antioxidant capacity of these organs. Given the high berberine content of the root (6.26 mg g^-1^ Dry Weight), and after trying to find a simple yet effective method to extract berberine, it was found that 80% ethanol containing 2% acetic acid at 25 °C with 72 h of maceration gave the highest berberine yield. Overall, the distribution and accumulation patterns of the secondary metabolites in the different organs of *B. integerrima* lead to their different applications.

## 1 Introduction

The genus *Berberis*, of which 500 species are distributed worldwide, belongs to the family of Berberidaceae. Various parts of this plant are used in the food, dyeing and pharmaceutical industries [1,2]. Barberry is a thorny and woody shrub with yellow flowers and wood, having medicinal and edible properties due to its secondary metabolites [3]. Previous studies have reported that different barberry species contain a wide range of phytochemicals such as alkaloids, flavonoids, saponins, anthocyanins, tannins, carbohydrates, proteins, lipid, fiber and vitamins, which are distributed in fruit, leaf, stem and root organs with different patterns [4]. Khromykh et al. [5] reported a positive correlation between phenolics contents and antioxidant capacity of the fruits of 5 barberry species (*B. vulgaris*, *B. amurensis*, *B. canadensis*, *B. koreana* and *B. declinata*). It was also shown that the fruit of *B. libanotica* contains more phenolic compounds than its leaf, resulting in a stronger antimicrobial activity of the fruit extract [6]. In a more comprehensive research, 33 compounds including various alkaloids, flavonoids, simple phenolics and esters were found in the *B. iliensis* fruit, leaf and root, making these organs potent antioxidant and antimicrobial agents [7]. Besides, recent studies have focused on the identification of individual phenolic compounds such as rutin, quercetin, luteolin, chlorogenic acid, gallic acid, catechin, vanillic acid, caffeic acid, syringic acid, *P*-coumaric acid and ferulic acid in the different organs of barberry [8,9]. Nevertheless, further studies are still needed on the quantity of these compounds in different parts of various species of this plant.

As one of the most important groups of the secondary metabolites, phenolic compounds are widely present in various plant species. They range in diversity from simple phenolics to complex polymers such as flavonoids and anthocyanins. These compounds are synthesized via the phenylpropanoid pathway, in which phenylalanine ammonia lyase (PAL) and tyrosine ammonia lyase (TAL) act as the first and key regulatory enzymes. All types of phenolic compounds, especially flavonoids, have the ability to scavenge free radicals due to their structural properties and are therefore called antioxidants [10,11]. In addition, these compounds also have anti-inflammatory, anti-bacterial and anti-fungal effects, which significantly increase their importance in the food and pharmaceutical industries [12]. So, these chemicals’ contents and diversity, varying according to species and tissue, could be one of the decisive factors for the quality and value of the plants in the food and pharmaceutical industries [13].

Isoquinoline alkaloids such as berberine, palmatine, columbamine, jatrorrhizine and epiberberine are another class of phytochemicals in *Berberis*, making this genus valuable [14–16]. Among isoquinoline alkaloids found in barberry, berberine is known one of the most important alkaloids in various organs, especially the root, and has been the subject of many studies in recent years. Several reports have shown that berberine, as an antioxidant compound, plays an effective role in preventing and treating diabetes, cancer, inflammation and microbial infections [17–20].

Given the value of this compound, it is very important to identify cost-effective methods to purify berberine or increase its amount in the plant extract. There are different procedures to optimize the extraction of plant-based biocompounds. Some studies show that the type of solvent, its acidity, time and temperature are effective factors during the extraction and purification process [21]. Babu et al. [22] reported that temperature has a crucial role in berberine concentration of *Coscinium fenestratum* stem extract. They showed that the berberine content is higher in samples extracted at low temperatures. One study indicated that the effect of inorganic acids is greater than organic acids in the extraction of berberine from *Coptis chinensis* rhizomes. It was observed that 0.34% phosphoric acid gives the highest yield [23]. Wu et al. [24] found that four variables, including solvent concentration, pH, liquid-material ratio and extraction time, have significant effect on the concentration of anti-tumor alkaloids extracted from *B. amurensis* stem. An investigation on *B. integerrima* has also indicated that berberine concentration in stem extract is dependent on the solvent percentage, temperature and time of extraction [21]. However, the simultaneous effect of all the factors involved in optimal berberine extraction still needs to be further investigated.

*B. integerrima* Bunge is one of the species of *Berberis* genus whose medicinal value has been studied in recent years [25–27]. However, little information is available on the metabolic composition of various organs and tissues of this herb. Therefore, in our study, we compared the contents of various compounds such as alkaloids (berberine), phenolic acids, flavonoids, anthocyanin, the activity of PAL and TAL enzymes as well as the antioxidant capacity of different organs and tissues of this barberry species. Besides, the effect of different parameters including solvent type, solvent ratio, solvent acidity, maceration time and temperature was evaluated on the efficiency of berberine extraction and purification from *B. integerrima* root.

## 2 Materials and methods

### 2.1 Collection and preparation of plant materials

Aerial parts and roots of *B. integerrima* were collected from wild plants in Mazandaran region (36º35′14ʺ N, 53º50′34ʺ E, and 1025 m above sea level) in April 2022. The permission to harvest this plant was provided by the Research Ethics Committee of Tarbiat Modares University. The formal identification of the *B. integerrima* was carried out by Dr. Shahrokh Kazempour-Osaloo, professor of plant systematic. A voucher specimen of *B. integerrima* (No. 0012) was preserved in the Herbarium of Department of Plant Biology of Tarbiat Modares University. Fruit, leaf, stem, root, stem bark, root bark, stem pith and root pith were separated and used for further analysis. First, the samples were dried in oven at 40 ºC. Then, the dried samples were ground into powder with a grinder and stored in dark containers. To measure enzyme activity and antioxidant capacity, the fresh plant organs were immediately brought to the laboratory and then frozen with liquid nitrogen and kept at -20 °C.

### 2.2 Determination of phenolic compounds

To measure total phenolic compounds, 0.1 g of the powdered samples was homogenized in 3 mL of 1% (v/v) acidic methanol according to Ainsworth and Gillespie [28]. Then, the methanolic extract was used to determine the contents of total phenolic, flavonoid and flavonol.

#### 2.2.1 Total phenolic content.

Total phenolic content was determined according to Ainsworth and Gillespie [28] with some modifications. 5 mL of Folin-Ciocalteu solution (10%, v/v) and 4 mL of sodium carbonate (7.5%, w/v) were added to 1 mL of methanolic extract. After 2 h, the absorbance of the samples was evaluated at 730 nm. The standard curve of gallic acid was used to determine the total phenolic content (S1 Fig).

#### 2.2.2 Total flavonoid content.

To analyze the content of total flavonoid according to Chang et al. [29], 0.5 mL of methanolic extract was first added to 100 µL of 1 M potassium acetate and 100 µL of 10% (w/v) AlCl_3_. Then, 1.5 mL of methanol (80%, v/v) and 2.8 mL of deionized water were added to the reaction mixture. All samples were kept in the dark for 30 min. After that, the absorbance was measured at 450 nm. The rutin standard curve was used to measure the total flavonoid content (S2 Fig).

#### 2.2.3 Total flavonol content.

The content total flavonol, as one of the subgroups of flavonoids, was evaluated using the method of Akkol et al. [30] with a slight modification. 0.5 mL of methanolic extract was combined with 1.5 mL of 5% (w/v) sodium acetate and 0.5 mL of 2% (w/v) AlCl_3_. The absorbance of reaction mixture was read at 440 nm after 2 h. The rutin standard curve was used to ascertain the total flavonol content (S3 Fig).

#### 2.2.4 Total anthocyanin content.

To extract total anthocyanin, dried plant powder (0.1 g) was homogenized with 5 mL of acidic methanol (containing 1% HCl). The extracts were left overnight in the dark. Finally, the absorbance of the samples was measured at 550 nm using a spectrophotometric method [31]. The anthocyanin concentration was calculated using the extinction coefficient of 0.033 cm^-1^ µM^-1^.

### 2.3 Quantification of phenolic acids by HPLC

The measurement of individual phenolic acids was performed according to the method of Owen et al. [32]. Dried plant materials (0.2 g) were mixed with 3 mL of methanol. The extract was placed in an ultrasonic bath (Bandelin, Berlin, Germany) for 30 min and then placed on a shaker at 100 rpm for 3 h. After centrifugation of the samples, the supernatants were transferred to another tube for air drying. The residue was added with 4 mL of acetonitrile and 3 mL of n-hexane and the lower phase was exposed to air to dry. Finally, 500 µL of methanol (HPLC grade) was used to prepare samples to be injected into the HPLC instrument (Agilent Technologies 1260 infinity, USA) equipped with a C18-ODS3 column (5 μm, 250 × 4.6 mm) and a dual-array-diode (DAD) detector. The mobile phase consisted of 2% aqueous acetic acid (A) and methanol (B) with a gradient system as followed: 0–2 min 95% A, 2–8 min 95% A, 8–10 min 75% A, 10–20 min 60% A, 20–30 min 50% A, 30–40 min 0% A, 40–50 min 95% A. The detector was set at 278 and 300 nm, and the flow rate was 1 mL min^-1^ [33]. The quantitative analysis was performed for 3 biological replicates. Individual phenolic acids were identified based on the retention times of their reference purchased from sigma-Aldrich. Quantification of gallic acid, caffeic acid, ferulic acid and cinnamic acid in the extracts was also performed using the external calibration method. HPLC chromatogram and standard curves of the identified phenolic acids have been shown in S4 and S5 Figs.

### 2.4 Quantification of flavonoids by HPLC

To identify individual flavonoids by HPLC, 0.2 g of dried plant powder was mixed with 3 mL of methanol (40%, v/v), including 0.5% (v/v) acetic acid. After 12 h of shaking and centrifugation (10 min, 10000 × *g*), the supernatant was analyzed by HPLC. The mobile phase consisted of 0.5% phosphoric acid in deionized water (A) and acetonitrile (B). The UV detector was set at 254, 280, 300 and 350 nm [34]. The gradient program was as follows: 0–30 min 82% A, 30–60 min 33% A, 60–65 min 82% A, 65–70 min 82% A. Chromatography was carried out at 25 ºC with a flow rate of 0.8 mL min^-1^ [33]. Flavonoids were recognized based on the retention times of their references for 3 biological replicates. The external calibration method was used for quantification of catechin, resveratrol and luteolin. HPLC chromatogram and standard curves of the identified flavonoids have been shown in S6 and S7 Figs.

### 2.5 Phenylalanine ammonia-lyase and tyrosine ammonia-lyase enzyme activities

To determine the activity of key enzymes involved in phenolics biosynthesis, soluble proteins were extracted according to Bradford [35] method. For this, 0.2 g of fresh sample was homogenized in 2 mL of 50 mM potassium phosphate buffer (pH 7.0). The solution was centrifuged (12,000 × *g*, 20 min, 4 °C) and the supernatant was used to assay the enzyme activities. The activity of PAL and TAL enzymes were measured by monitoring the formation of cinnamic acid and *p*-coumaric acid, respectively [36]. For preparing reaction solution, 150 µL of protein extract was added to 200 µL of L-phenylalanine (0.1 M) (TAL: L-tyrosine 0.1 M) and 650 µL of potassium phosphate buffer (0.1 M, pH 8.0). The solution was then incubated at 37 °C and 30 °C, as optimal temperatures of PAL and TAL activities, respectively, for 1 h. After that, the reaction was stopped by adding 50 µL of HCl (6 N). The solution was extracted three times with 500 µL of ethyl acetate. After evaporation of the separated ethyl acetate phase, the residue was dissolved in 1 mL of NaOH (0.05 M). Finally, the absorbance of produced cinnamic acid and *p*-coumaric acid was read at 290 and 310 nm, respectively. Total protein concentration was determined using the bovine serum albumin standard curve. The enzyme activities were calculated using a standard curve and expressed in μmol of cinnamic acid or *p*-coumaric acid mg protein^-1^ h^−1^ (S8 Fig).

### 2.6 Determination of total alkaloid content

The extraction and measurement of the total alkaloid were performed according to the method of Shamsa et al. [37]. Dried plant powder (0.2 g) was mixed with 3 mL of methanol and placed on a shaker at 100 rpm for 24 h. After the centrifugation of obtained solution, the supernatant was transferred to a tube and placed in an oven at 45 ºC for evaporation. To the dried extract, 5 mL of HCl (2 N) was added, followed by centrifugation. 1 mL of the resulting solution was washed three times with 10 mL of chloroform. The pH of the solution was adjusted to 12.7 with NaOH (6 M). To the alkaline solution were added 5 mL of bromocresol green (BCG) solution and potassium phosphate buffer (pH 4.7). The reaction mixture was shaken, and 10 mL of chloroform was added to the mixture. Extraction was carried out under vigorous shaking. Finally, the chloroform phase was collected and its absorbance was read at 470 nm by a spectrophotometer. The standard curve of berberine was used to determine the total alkaloid content in mg g^-1^ DW (S9 Fig).

### 2.7 Quantification of berberine by HPLC

Dried powder of different organs (0.2 g) blended with 3 mL of acidic ethanol (80% v/v) and stored at room temperature in the dark for 2 h. The plant extract was centrifuged (12,000 × *g*, 10 min) and 1 mL of supernatant was poured into a microtube and dried at room temperature. Then, 500 µL of methanol (HPLC grade) was added to the dried residue and injected into the HPLC device (Agilent Technologies 1260 infinity, USA). The stationary phase consisted a C18-ODS3 column (5 μm, 250 × 4.6 mm) and the detector was a photodiode array. The elution solvents were also acidic deionized water (A) and acetonitrile (B) mixed in a gradient of 0–5 min 90% A, 5–10 min 60% A, 10–15 min 20% A, 15–25 min 90% A, with a flow rate of 1 mL min^-1^. The detector was set at 266 nm for berberine detection [38]. Berberine was recognized based on its reference retention time and the external calibration method was used for its quantification. The relevant HPLC chromatograms and standard curve have been shown in S10 and S11 Figs.

### 2.8 Antioxidant capacity

To investigate the antioxidant activity, 0.1 g of frozen sample was homogenized with 6 mL of ice-cold methanol. Subsequently, all extracts were centrifuged at 5,000 × *g* for 10 min at 4 °C and used for antioxidant assays [39,40].

#### 2.8.1 DPPH free radical-scavenging activity.

This test was performed according to the procedure of Maikai et al. [39] procedure. 1 mL of 0.002% DPPH solution was combined with 1 mL of methanolic extract and then kept in the dark for 30 min. The absorbance of samples was read at 517 nm by the spectrophotometer. The DPPH free radical-scavenging activity was calculated using the following formula:

%DPPH scavenging= ((control absorbance – extract absorbance)/ (control absorbance)) ×100

#### 2.8.2 Ferric reducing antioxidant power (FRAP).

The FRAP assay was developed by Lim et al. [40]. 1 mL of methanolic extract was added to 2.5 mL of potassium phosphate buffer (0.2 M, pH 6.6) and 2.5 mL of 1% potassium ferricyanide (K_3_Fe (CN)_6_). The reaction mixture was incubated at 50 °C for 30 min. Then, 2.5 mL of 10% trichloroacetic acid (TCA) was added and the resulting solution centrifuged at 5,000 × *g* for 10 min. At the end, 2.5 mL of deionized water and 0.5 mL of 0.1% ferric chloride (FeCl_3_) were added to 2.5 mL of the supernatant and the absorbance was evaluated at 700 nm. The gallic acid standard curve was used to calculate the ferric reducing antioxidant power (S12 Fig).

### 2.9 Investigating optimal conditions for berberine extraction

To determine the optimal conditions for berberine extraction from *B. integerrima* root, the effects of different factors including solvent type, solvent ratio, solvent acidity, maceration time and temperature were evaluated. First, the effect of different solvents was assessed on the concentration of extracted berberine. For this, an equal amount of root powder (1.5 g) was macerated separately in 50 mL of water, methanol, ethanol, acetone, propylene alcohol, and ethylene glycol. The content of extracted berberine was then determined by HPLC according to the method of Srinivasan et al. [38]. Next, the effect of solvent ratio (60, 70, 80 and 90%) was examined. Furthermore, the effect of different concentrations of acetic acid (0.5, 1, 2 and 4%) on the extraction of berberine was determined. Finally, the effects of maceration time (24, 72 and 168 h) and temperature (25, 40 and 60 ºC) on the amount of this alkaloid in the root extract were also investigated.

### 2.10 Purification of berberine

To purify berberine from *B*. *integerrima* root*,* 1 mL of ethanolic extract, prepared using optimal method obtained from our previous experiment, was injected into a reversed-phase preparative HPLC with a C18 column (7 μm, 21.2 × 150 mm), and a flow rate of 10 mL min^-1^. The gradient mobile phase contained 0.05% ortho phosphoric acid in water (A) and acetonitrile (B), and the UV detector was set at 266 nm. The elution was performed as follows: 1–2 min 90% A, 2–4 min 75% A, 4–5 min 65% A, 5–10 min 60% A, 10–15 min 20% A, 15–20 min 90% A. The resulting fraction was collected and dried. LC-ESI-MS and MS/MS in positive ion mode was then used to confirm the accuracy of the purified fraction. A liquid chromatography (Waters Alliance 2695) coupled to a single quadrupole ion mass spectrometer equipped with a C18 column (2.1 × 150 mm, 3 µm; Waters, USA) was used. The binary mobile phase comprised ACN + 0.1% formic acid (50%) (A) and water + 0.1% formic acid (50%) (B). The column temperature was maintained at 40 °C, the flow rate and the injection volume were 0.2 mL min^-1^ and 2 µL, respectively. The spectra were recorded in the targeted mode within the m/z mass range of 100–700 (S13 Fig).

### 2.11 Statistical analysis

All experiments were performed in three independent replicates and statistically analyzed using SPSS 24 software. Duncan multiple range test was performed to determine statistical differences between different groups. A *p*-value less than 0.05 was considered for a significant difference. Furthermore, the data were subjected to Hierarchical Cluster Analysis (HCA) analysis using the online metabolomics data processing tool MetaboAnalyst (http://www.metaboanalyst.ca).

## 3 Results

### 3.1 Total phenolic compounds in different organs and tissues

#### 3.1.1 Total phenolic content.

**Fig 1:**
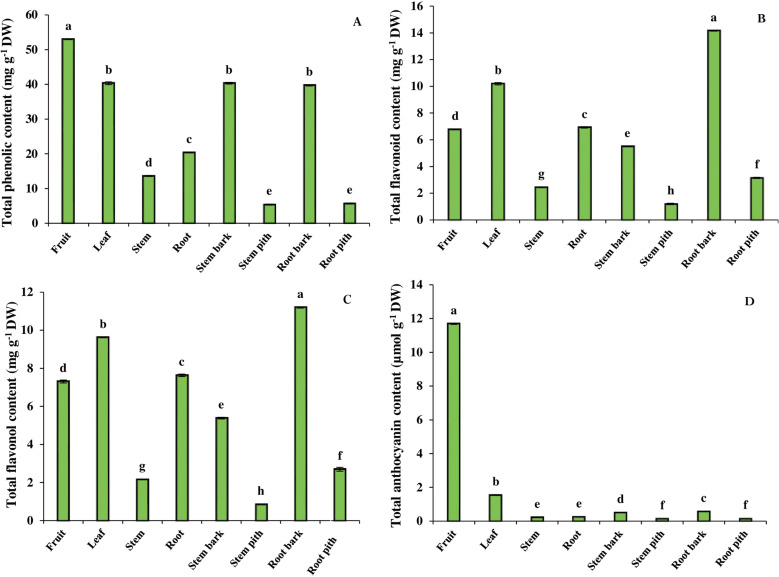
The contents of total phenolic (A), total flavonoid (B), total flavonol (C), and total anthocyanin (D) in different organs and tissues of *B. integerrima*. Data are means ± SE. Significant differences (*p* ≤ 0.05) are indicated by different letters.

As shown in Fig 1A, the total phenolic content was the highest in the fruit, and then exhibited a decreasing trend in the leaves, roots and stems, respectively. The total phenolic content of fruits was found to be 1.3 times higher than that of leaves, while it was 2.6 and 3.9 times higher than that of root and stem, respectively. Tissue comparison also showed that the content of total phenolic in root and stem bark was significantly higher than that of their pith. There was no significant difference between the root and stem bark and their pith tissue, where the bark of these organs had approximately 7 times more total phenolic than the pith tissue.

#### 3.1.2 Total flavonoid content.

The total flavonoid content varied in the different organs and tissues of *B. integerrima*. As shown in Fig 1B, the highest level of total flavonoid was found in the leaf and then showed a decreasing trend in the root, fruit and stem. The total flavonoid value in the leaf was 31.6%, 33.6% and 76.2% higher than that in the root, fruit and stem, respectively. In addition, the highest amount of total flavonoid was found in the root bark (14.14 mg g^-1^ DW) and the lowest was observed in the stem pith (1.19 mg g^-1^ DW).

#### 3.1.3 Total flavonol content.

The determination of total flavonol content showed that the variations in the amount of these compounds followed a similar pattern to the total flavonoid content in different organs and tissues*.* The highest content was found in leaf, then in root, fruit and stem organs, respectively. Furthermore, bark of root and stem had the highest total flavonol content compared to their pith tissue (Fig 1C).

#### 3.1.4 Anthocyanin content.

The concentration of anthocyanin in the fruit differed significantly from that in other organs, so that it was about 7.7 times higher than in the leaf. The lowest content of anthocyanin was obtained in the root and stem, and these organs did not show a significant difference. In the comparison of tissues, it was also found that the greatest amount of these colored compounds accumulated in the root and stem bark (Fig 1D).

### 3.2 The contents of phenolic acids

The accumulation of individual phenolic acids in different organs and tissues of *B. integerrima* was investigated by HPLC and four phenolic acids including caffeic acid, gallic acid, ferulic acid and cinnamic acid were quantified (Table 1). The highest amount of caffeic acid was detected in the fruit organ (16.63 µg g^-1^ DW), which was 8.3 times higher than that of the stem. This phenolic acid was not observed in the leaf and root organs. Examination of the caffeic acid content in the stem tissue showed that its amount in the bark was 3 times than the pith. The amount of gallic acid in the stem was 8.3 times higher than in the root, while its content was the lowest in the leaf and fruit. On the other hand, the highest amount of ferulic acid was observed in the root (112.02 µg g^-1^ DW) and then in the stem (39.18 µg g^-1^ DW). Its concentration was also higher in the bark tissue than in the pith of these organs. The amount of this phenolic acid in leaf and fruit had no significant difference. The determination of cinnamic acid showed that the leaf contained a greater amount of this compound than the root (6.7 times), fruit (9 times) and stem (13.5 times). The accumulation of this phenolic acid in the bark tissue of the stem and root was higher than in the pith, and it was not detected in the pith tissue of the root.

**Table 1:** The contents of phenolic acids in different organs and tissues of *B. integerrima.*

Organs and Tissues	Phenolic acids			
	Gallic acid (µg g^-1^ DW)	Caffeic acid (µg g^-1^ DW)	Ferulic acid (µg g^-1^ DW)	Cinnamic acid (µg g^-1^ DW)
Fruit	2.19 ± 0.25^e^	16.63 ± 1.29^a^	1.58 ± 0.06^d^	0.34 ± 0.02 cd
Leaf	4.40 ± 1.08^e^	ND	2.95 ± 0.02^d^	2.70 ± 0.06^a^
Stem	63.81 ± 9.51^b^	2.02 ± 0.47^c^	39.18 ± 3.59^c^	0.24 ± 0.01^d^
Root	7.61 ± 0.60^d^	ND	112.02 ± 1.64^a^	0.40 ± 0.04 cd
Stem bark	144.50 ± 1.83^a^	9.25 ± 0.51^b^	67.78 ± 0.14^b^	1.11 ± 0.03^b^
Stem pith	14.12 ± 0.15^d^	3.06 ± 0.08^c^	67.80 ± 6.41^b^	0.44 ± 0.11^c^
Root bark	41.87 ± 8.005^c^	3.56 ± 0.01^c^	119.26 ± 10.25^a^	0.26 ± 0.01^d^
Root pith	1.81 ± 0.08^e^	ND	86.58 ± 18.18^b^	ND

Data are means ± SE. Significant differences (*p* ≤ 0.05) are indicated by different letters.

### 3.3 The contents of flavonoids

The concentration of resveratrol, luteolin and catechin was higher in the root compared to the other organs. Catechin was the most abundant flavonoid in the root with 2420.7 µg g^-1^ DW, the amount of which was 14.2 times and 366.7 times higher than that of resveratrol and luteolin, respectively. Tissue comparison also showed a greater accumulation of catechin in the stem and root bark compared to the pith tissue. The resveratrol content in the leaf and fruit was very low and they did not differ significantly. This flavonoid compound was not detected in the stem. The inter-tissue comparison showed that the amount of resveratrol was much higher in the root bark than in the root pith. On the other hand, the amount of luteolin was the same in the fruit and stem organs. This flavonoid compound was not found in the leaf and stem pith (Table 2).

**Table 2:** The contents of flavonoids in different organs and tissues of *B. integerrima.*

Organs and Tissues	Flavonoids		
	Catechin (µg g^-1^ DW)	Resveratrol (µg g^-1^ DW)	Luteolin (µg g^-1^ DW)
Fruit	153.14 ± 31.76^e^	2.71 ± 0.09^c^	1.78 ± 0.11^c^
Leaf	545.03 ± 26.64^d^	3.81 ± 0.82^c^	ND
Stem	1053.37 ± 71.92^c^	ND	2.47 ± 0.15^c^
Root	2420.72 ± 68.02^b^	170.31 ± 23.45^a^	6.63 ± 1.12^a^
Stem bark	5016.50 ± 157.47^a^	2.47 ± 0.78^c^	3.95 ± 0.83^b^
Stem pith	190.97 ± 7.39^e^	ND	ND
Root bark	2638.75 ± 131.68^b^	104.22 ± 7.98^b^	4.75 ± 0.43^b^
Root pith	106.46 ± 34.15^e^	9.46 ± 1.18^c^	1.82 ± 0.23^c^

Data are means ± SE. Significant differences (*p* ≤ 0.05) are indicated by different letters.

### 3.4 Phenylalanine ammonia-lyase and tyrosine ammonia-lyase enzyme activities

**Fig 2:**
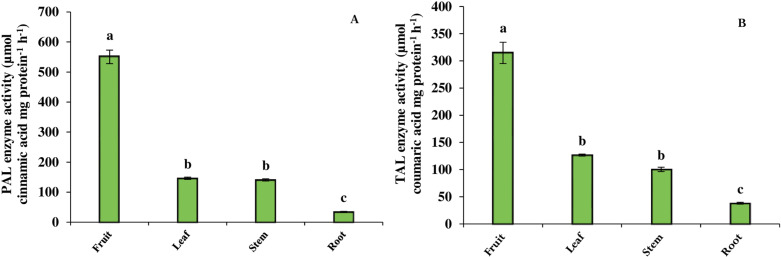
The activity of PAL (A) and TAL (B) enzymes in different organs of *B. integerrima*. Data are means ± SE. Significant differences (*p* ≤ 0.05) are indicated by different letters.

The investigation of PAL and TAL enzymes as the key regulatory factors in the production of phenolic compounds showed that their activities followed a similar pattern, being more active in the aerial parts compared to the root. The maximum activity of PAL and TAL occurred in the fruit, which was 93.7% and 87.8% higher than that of the root, respectively (Fig 2A and B).

### 3.5 Total alkaloid content

**Fig 3:**
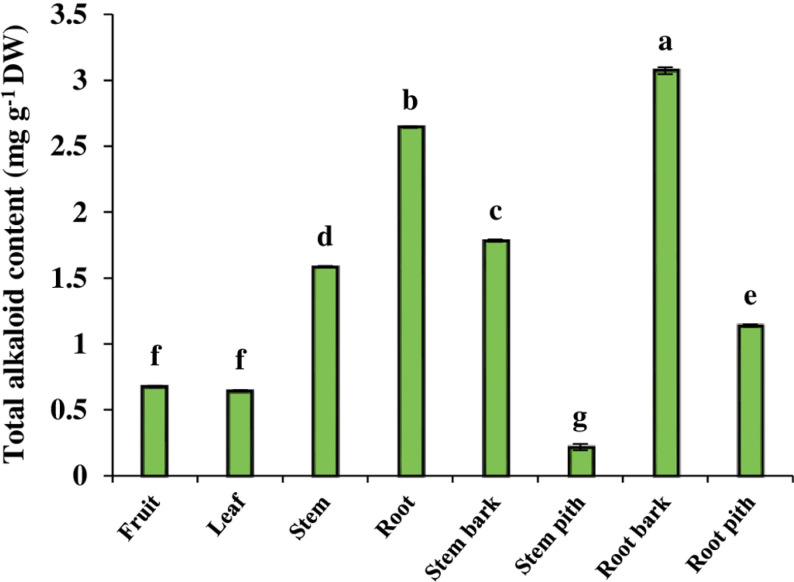
The content of total alkaloid in different organs and tissues of *B. integerrima.* Data are means ± SE. Significant differences (*p* ≤ 0.05) are indicated by different letters.

The content of total alkaloid, the most important secondary metabolites in *B. integerrima*, had significant differences in the various organs and tissues. Of the different organs, the highest content of total alkaloid was observed in the root, while the leaf and fruit showed the lowest content of total alkaloid. So that its content in the root was about 42.3% higher than the stem and 76.9% higher than the leaf and fruit. When comparing the tissues, it was found that the content of total alkaloid in the root bark and stem bark was higher than in the pith tissue. In addition, the total alkaloid content of the root bark was 43.3% higher than that of the stem bark (Fig 3).

### 3.6 The content of berberine

**Fig 4:**
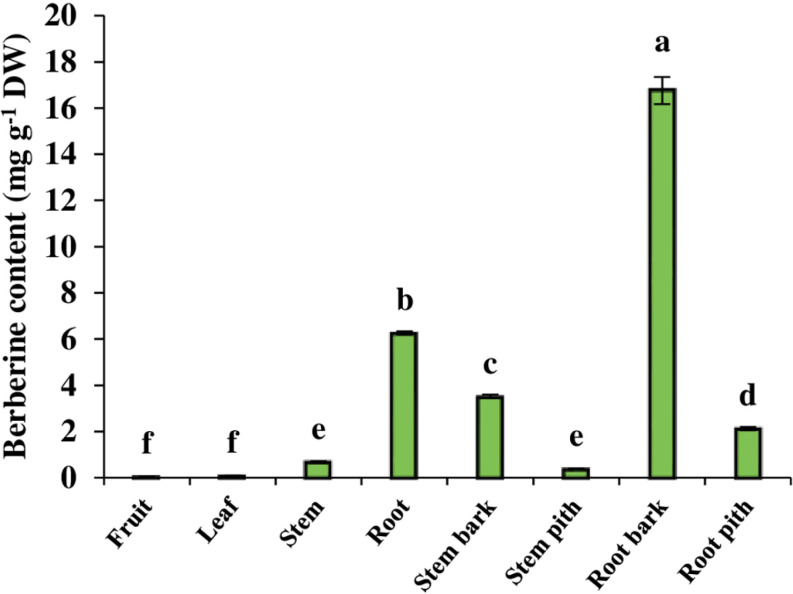
The content of berberine in different organs and tissues of *B. integerrima.* Data are means ± SE. Significant differences (*p* ≤ 0.05) are indicated by different letters.

The amount of berberine, one of the most important alkaloids of barberry, was the highest in the root and the lowest in the leaf and fruit. Berberine content was 6.26 mg g^-1^ DW in root, which was 10.3, 78. 25 and 208.6 times higher than in the stem, leaf and fruit, respectively. Furthermore, there was no significant difference between leaf and fruit. The comparison of different root and stem tissues showed that the highest berberine content was found in the root bark and the lowest content observed in the stem pith. The amount of berberine in the root bark was 4.7 times higher than in the stem bark and 7 times higher in the root pith than in the stem pith (Fig 4).

### 3.7 Antioxidant capacity of different organs

**Fig 5:**
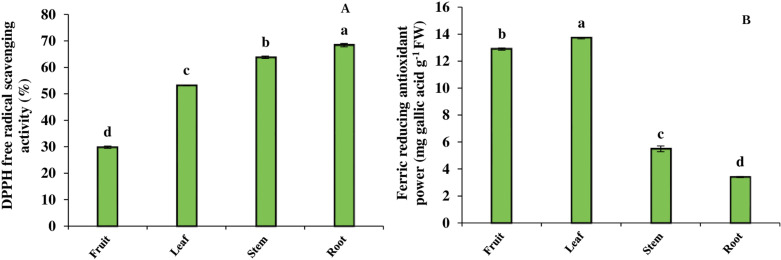
DPPH free radical-scavenging activity (A) and ferric reducing antioxidant power (FRAP) (B) in different organs of *B. integerrima.* Data are means ± SE. Significant differences (*p* ≤ 0.05) are indicated by different letters.

According to Fig 5A, the free radical-scavenging activity of DPPH in different organs showed significant differences. The highest radical-scavenging activity was measured in the root (68.39%), followed by the stem with 63.78%, the leaf with 53.18% and the fruit with 29.84% inhibition. In contrast, the ferric reducing antioxidant power (FRAP) was higher in the aerial parts than in the root. The highest ferric reducing power among the aerial organs was obtained in the leaf, so that this antioxidant capacity was 5.8% more than that of the fruit and 60.2% and 75% more than that of the stem and root, respectively (Fig 5B).

### 3.8 Optimization of berberine extraction

**Fig 6:**
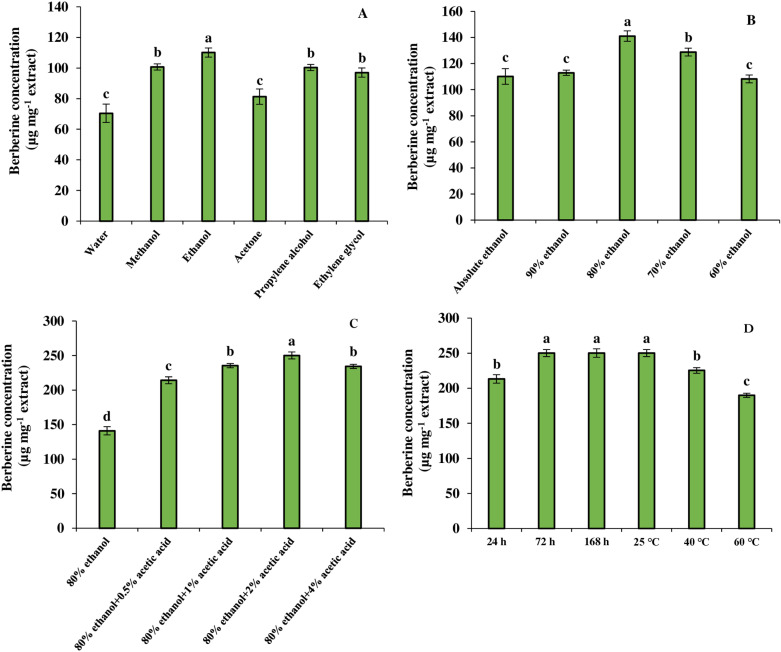
The effect of solvent type (A), solvent ratio (B), acid percentage (C), and temperature and maceration time (D) on the berberine extraction from *B. integerrima* root. Data are means ± SE. Significant differences (*p* ≤ 0.05) are indicated by different letters.

To determine the optimal condition for berberine extraction from *B. integerrima* root, different solvents were used and the results showed that extraction by ethanol had the highest berberine content (110.11 µg mg^-1^) (Fig 6A). Methanol, propylene alcohol and ethylene glycol extracted the same amount of berberine (approximately 100 µg mg^-1^), and water and acetone extracted the least amount of berberine (about 70 µg mg^-1^). The effect of ethanol percentage (as the best solvent) on the berberine extraction was also investigated and 80% ethanol was found to be the most efficient in extracting 141.09 µg berberine in 1 mg of extract. Interestingly, the higher and lower proportion of ethanol resulted in a decrease in the concentration of extracted berberine (Fig 6B). Using different concentrations of acetic acid also had an effect on the amount of berberine extracted. As shown in Fig 6C, the addition of 2% acetic acid to 80% ethanol resulted in a 43.6% increase in the berberine extraction to 250.13 µg mg^-1^ extract. Similar to the change in the percentage of ethanol, other proportion of acetic acid led to a decrease in the concentration of berberine. Finally, temperature and maceration time were found to have a significant effect on the concentration of extracted berberine. Increasing the maceration time up to 72 h caused an enhancement in berberine extraction. In addition, increasing the temperature resulted in a decrease in the efficiency of this alkaloid extraction (Fig 6D).

### 3.9 Purification of berberine

**Fig 7:**
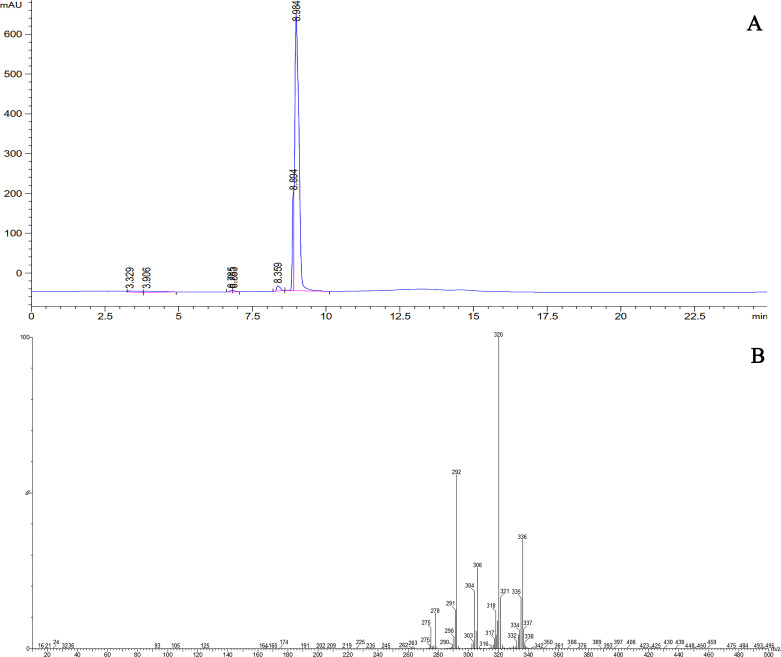
The analytical HPLC chromatogram (A) and LC-MS/MS spectrum (B) of berberine purified from *B. integerrima* root.

After determining the optimal method for extracting berberine, we attempted to purify this valuable alkaloid by a preparative HPLC. The fraction isolated by the preparative HPLC was injected into analytical HPLC and its peak was analyzed. As shown in Fig 7A, it appears that the isolated fraction contains berberine. To confirm this idea, LC-ESI-MS and MS/MS analysis were performed and the results verified that the fraction was pure berberine (Fig 7B).

### 3.10 Visualization of data by hierarchical cluster analysis

**Fig 8:**
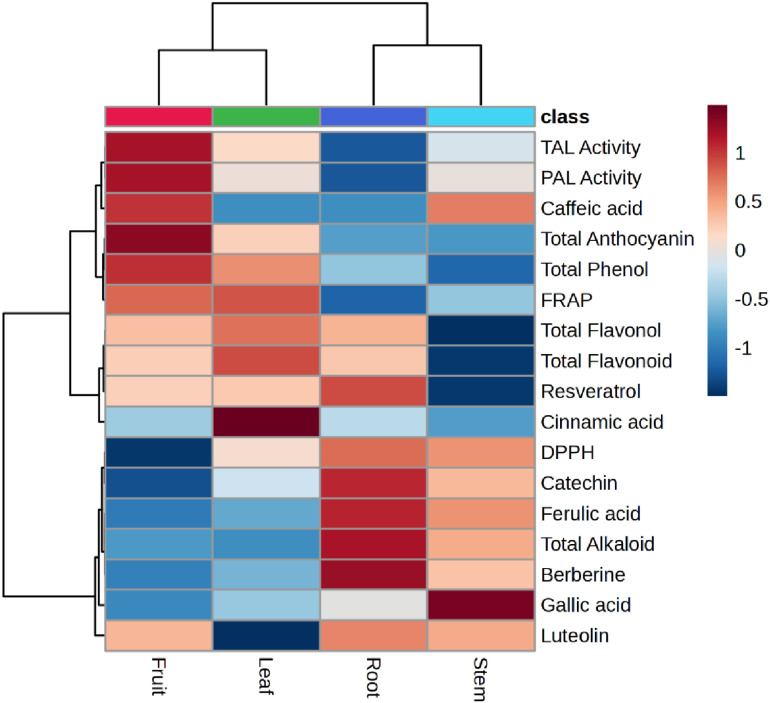
Hierarchical cluster analysis was used for clustering of different metabolites in various organs of *B. integerrima* based on Pearson correlations coefficient. The blue cells show strong negative and the red cells are the sign of strong positive correlation.

To better understand the differences in the distribution of different metabolites in the various organs of *B. integerrima*, we visualized the data using Hierarchical Cluster Analysis (HCA). The metabolites evaluated in different organs were classified into 3 clusters (1–3), where each cluster represents similar changes in metabolites of these organs. As shown in Fig 8, it can be stated that most of the phenolic compounds, PAL, TAL enzymes and FRAP are classified in the same group, and show a positive correlation in the leaf and fruit organs. On the other hand, total alkaloid, berberine, some phenolics and DPPH are placed in another group with a positive correlation in stem and root.

## 4 Discussion

Due to its secondary metabolites, *B. integerrima* is a valuable plant in various industries and for the treatment of many diseases. The accumulation of various secondary metabolites in each organ of barberry has given it an important role in medicine. Therefore, in the current study, we attempted to investigate the distribution of various secondary metabolites in different organs and tissues of *B. integerrima.* The fruit, as the most consumed organ, plays an important role in regulating the immune system of our body due to its phenolic compounds [2]. Large number of studies have already been focused on phenolic compounds and their biological activities in plants. They have shown that these compounds have antioxidant activity [41]. One of the most important groups of phenolics in barberry fruit is anthocyanins, the high content of these compounds causes the color of this organ [42]. The results of the present study indicated that the content of total anthocyanin in the fruit of *B. integerrima* is 7.7 times higher than the leaf. In addition, we compared the contents of other groups of phenolic compounds in different organs and tissues of this barberry species and found the highest levels of total phenolic, total flavonoid and total flavonol occur in aerial parts. The results of investigations on *B*. *croatica* and *B. vulgaris* are also consistent with these observations [10]. In general, it can be concluded that the aerial organs of *B*. *integerrima* contain more phenolic compounds.

Further experiments provided more details on the individual phenolic acids and flavonoids in different organs and tissues of *B*. *integerrima*. HPLC results indicated the presence of four phenolic acids gallic acid, caffeic acid, ferulic acid and cinnamic acid at different levels in the fruit, leaf, stem and root. The most abundant phenolic acid detected in the fruit was caffeic acid with 16.63 µg g^-1^ DW, while its content in the stem, which had the highest value after the fruit, was 2.02 µg g^-1^ DW. On the other hand, gallic acid, ferulic acid and cinnamic acid were the most abundant acids in the stem, root and leaf, respectively. In addition, three flavonoids catechin, resveratrol and luteolin were detected in different organs and tissues of *B*. *integerrima* and interestingly, the highest amount of all was observed in the root. Recently, Dawra et al. [6] also identified 12 phenolic compounds in fruit and 8 phenolic compounds in leaf of *B*. *libanotica*, including gallic acid.

PAL and TAL enzymes are the entry point of primary metabolites to the phenylpropanoid pathway and are therefore important regulatory factors in the biosynthesis of phenolic compounds [43]. Variations in the activity of PAL and TAL enzymes among different organs of *B*. *integerrima* showed a similar pattern with the contents of phenolics, showing higher activity in the aerial parts of the plant than in the root, thus the maximum activity of PAL and TAL was evaluated in the fruit. Hierarchical clustering analysis (Fig 8) also confirmed the coordinated variations in phenolic compounds contents and PAL and TAL enzyme activities by placing them in the same cluster. In addition, this data visualization showed that phenolic compounds placed in the same group with red color, are positively correlated with leaf and fruit organs.

Alkaloids are another important group of secondary metabolites of *Berberis* genus, of which previous studies attributed some of the medicinal properties of barberry to the presence of these compounds [44]. In fact, barberry is known to be a rich source of various types of alkaloids with antioxidant and therapeutic properties [45]. Berberine is considered one of the main alkaloids of *Berberis* genus, which is why many studies have focused on this valuable alkaloid in various *Berberis* species. Garhwal [46], by studying *B*. *asiatica, B*. *aristata* and *B*. *lycium*, revealed that the berberine content of these three species is different and also their roots contain much more berberine compared to the stem. Other researchers have reported similar results and pointed out that the berberine content in the roots of various *Berberis* species is higher than in other organs [47]. It has been suggested that the higher berberine content in underground organs may be due to its sensitivity to the light [46,48]. Furthermore, since the antimicrobial and antifungal activity of this compound has been confirmed, the roots seem to use berberine as a defense agent [49, 50]. The results of current study displayed that the root of *B. integerrima* contained the highest amount of total alkaloid, 1.7 times more than the stem and 4.3 times more than the leaf and fruit. Given the variations in berberine content, our observations were similar to the previous studies and the root showed the highest level of this alkaloid. Since the berberine content of the root is approximately 90% higher than that of the stem and 99% higher than that of the leaf and fruit, this organ is the main source of berberine in *B. integerrima*. Accordingly, the clustering heatmap also classified total alkaloid and berberine in the same cluster with a red color in the root organ column (Fig 8).

As mentioned above, phenolic acids, flavonoids, anthocyanins and alkaloids act as non-enzymatic antioxidants. These compounds prevent oxidative damage to macromolecules and plant cell membranes by scavenging free radicals of reactive oxygen species (ROS) and reactive nitrogen species (RNS) [51]. In this study, different organs of *B. integerrima* showed different antioxidant capacities in Fe^3+^ reduction. It was found that the aerial parts, including leaf and fruit, had a greater capacity to reduce Fe^3+^ to Fe^2+^. Based on our observations, the aerial parts of *B*. *integerrima* contain high levels of phenolic compounds, and it is possible that these compounds play a role in regenerating the iron ions as antioxidant molecules. Wojdyło et al. [52], by examination on the contents of phenolics of 32 plant species and comparing their antioxidant capacity, reported that plant species with higher levels of phenolic compounds have higher antioxidant capacity. Additionally, there is another study showing that the contents of phenolic compounds in barberry fruit have a direct influence on its ability to reduce Fe^3+^ [53]. On the other hand, another experiment revealed that the root of *B*. *integerrima* has the highest inhibitory activity of DPPH free radical in comparison with aerial organs. Our results showed that DPPH free radical inhibition in the root was 68.39%, while it was 63.78% in the stem, 53.18% in the leaf and 29.84% in the fruit. Berberine, the main alkaloid of this barberry species, has strong antioxidant properties that may play a significant role in combating DPPH free radical. Previous studies demonstrated the ability of berberine to scavenge free radicals in a concentration-dependent manner. They also showed that berberine is as effective as ascorbic acid in eliminating DPPH free radical [17,54]. As shown in Fig. 8 there is a positive correlation between DPPH free radical-scavenging and berberine content in the root and stem organs, while FRAP and phenolic compounds also represent a positive correlation in the fruit and leaf organs.

**Fig 9:**
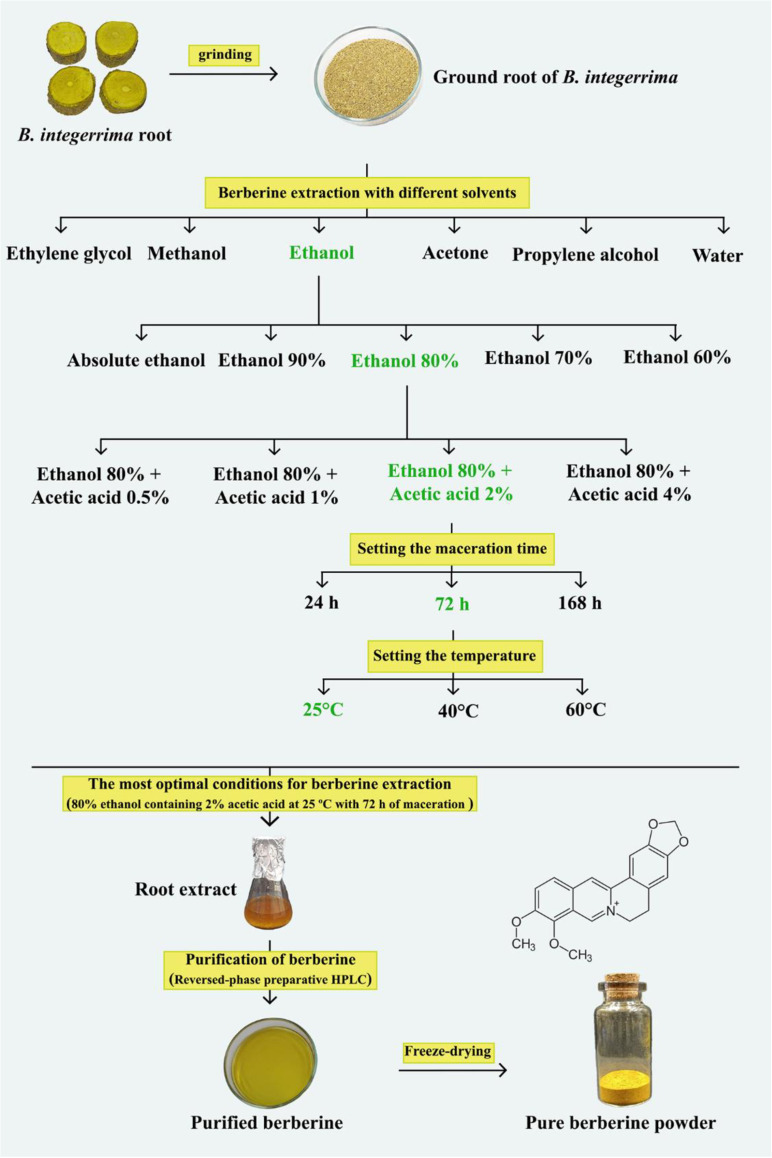
Schematic representation of optimization of berberine extraction from *B. integerrima* root.

Considering the importance of providing the maximum amount of for medicinal and industrial purposes, an attempt was made to develop a simple and efficient method for extracting this compound with high yield from the root of *B*. *integerrima*. Based on the results, ethanol had the highest extraction rate compared to other solvents. In addition, it was observed that the solvent ratio and acidity had a positive effect on the extraction rate of berberine, so that ethanol 80% combined with 2% acetic acid increased the extraction efficiency by 21.9% and 43.6%, respectively. A temperature of 25 ºC and 72 h of maceration were also the best conditions for berberine extraction (Fig 9). Subsequently, berberine was tried to purify from the extract obtained from *B*. *integerrima* root, which can be very valuable for special medical cases. Purified berberine was subjected to LC-MS and MS/MS, and the results were consistent with the spectral data recorded in previous studies [55].

## Conclusion

Overall, the distribution pattern of alkaloids and phenolic compounds in different organs of *B. integerrima* is different and some of these compounds are dominant in certain organs. It was found that the fruit of this plant contains the highest amount of total phenolic, total anthocyanin and caffeic acid. Total flavonoid and cinnamic acid were the dominant compounds in the leaf. Gallic acid showed its highest amount in the stem and finally the root had the highest amount of total alkaloid, berberine, ferulic acid, catechin, resveratrol and luteolin. It was also found that these organs have different antioxidant capacity depending on their specific compounds. Likewise, an optimal method was provided to improve the extraction efficiency as well as the purification of berberine from the root of *B. integerrima*.

## Supporting information

S1 Fig Total phenolic standard calibration curve. (PDF)

S2 Fig Total flavonoid standard calibration curve. (PDF)

S3 Fig Total flavonol standard calibration curve. (PDF)

S4 Fig Phenolic acids HPLC chromatogram of reference. (PDF)

S5 Fig Phenolic acids standard calibration curves. (PDF)

S6 Fig Flavonoids HPLC chromatogram of reference. (PDF)

S7 Fig Flavonoids standard calibration curves. (PDF)

S8 Fig PAL and TAL enzymes standard calibration curves. (PDF)

S9 Fig Total alkaloid standard calibration curve. (PDF)

S10 Fig Berberine HPLC chromatograms of (A) reference and (B) sample. (PDF)

S11 Fig Berberine standard calibration curve. (PDF)

S12 Fig FRAP standard calibration curve. (PDF)

S13 Fig Berberine mass spectrum. (PDF)

S1 Text Raw data. (XLSX)
