# Correction: A comparative study of alkaloid and phenolic compounds in different organs and tissues of *Berberis integerrima*

**DOI:** 10.1371/journal.pone.0333632

**Published:** 2025-09-30

**Authors:** The PLOS One Staff

## Notice of Republication

This article was republished on September 17, 2025, to correct an error in the formatting of the editor’s name and institution.

The publisher apologizes for the error. Please download this article again to view the correct version. The originally published, uncorrected article and the republished, corrected articles are provided here for reference.

## Supporting information

S1 File Originally published, uncorrected article. (PDF)

S2 File Republished, corrected article. (PDF)
