# Supplementary figures and images for: A comparative study of alkaloid and phenolic compounds in different organs and tissues of Berberis integerrima
Source: PLoS One. 2025 May 19;20(5):e0321255. doi: 10.1371/journal.pone.0321255 (PMC12087984; doi:10.1371/journal.pone.0321255)

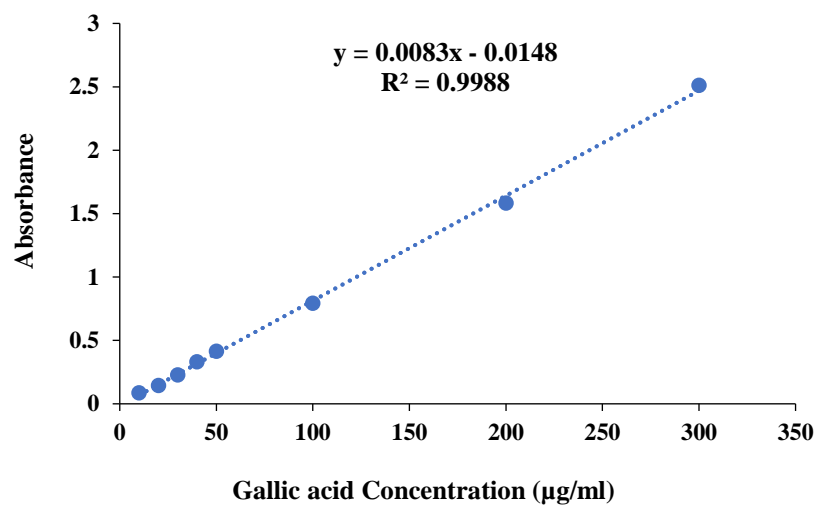

**S1 Fig.** Total phenolic standard calibration curve

Supplement: S1 Fig — (PDF) [file pone.0321255.s001.pdf]

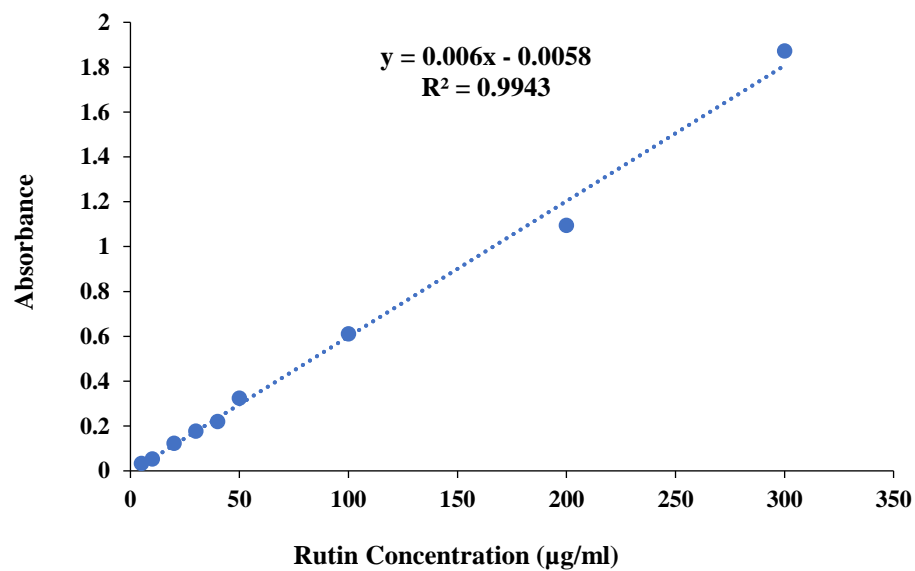

**S2 Fig.** Total flavonoid standard calibration curve

Supplement: S2 Fig — (PDF) [file pone.0321255.s002.pdf]

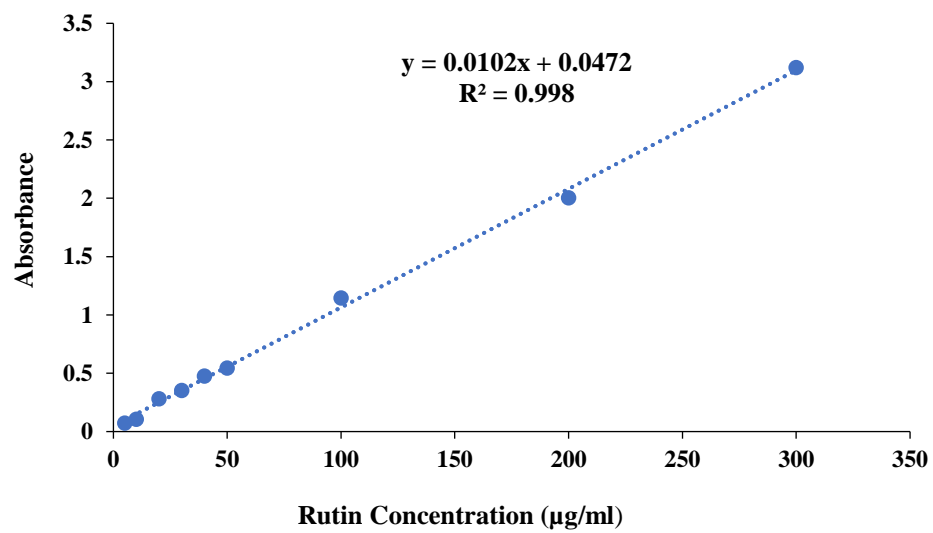

**S3 Fig.** Total flavonol standard calibration curve

Supplement: S3 Fig — (PDF) [file pone.0321255.s003.pdf]

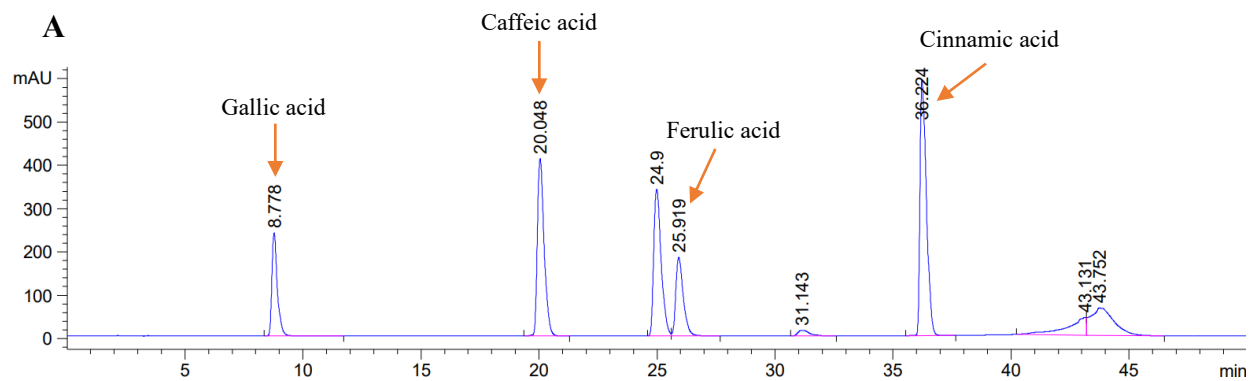

**S4 Fig.** Phenolic acids HPLC chromatogram of reference

Supplement: S4 Fig — (PDF) [file pone.0321255.s004.pdf]

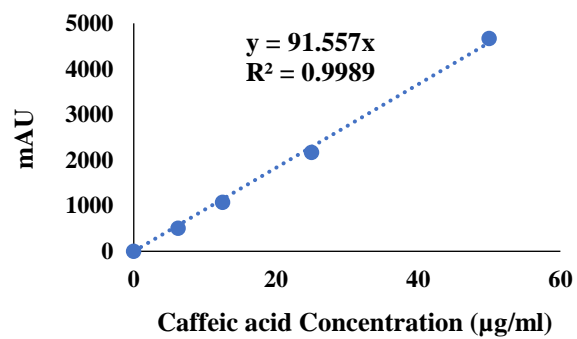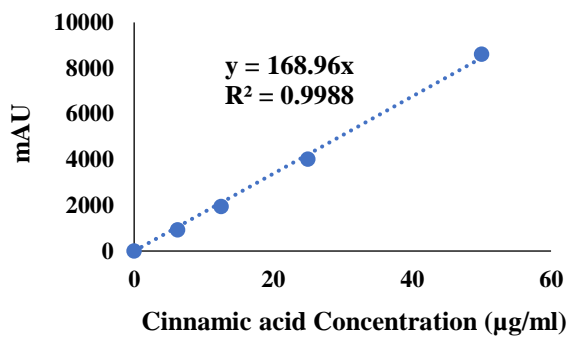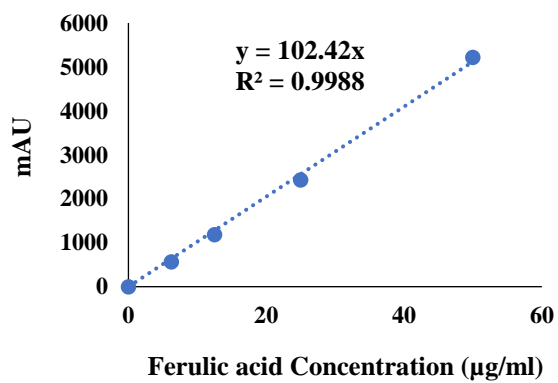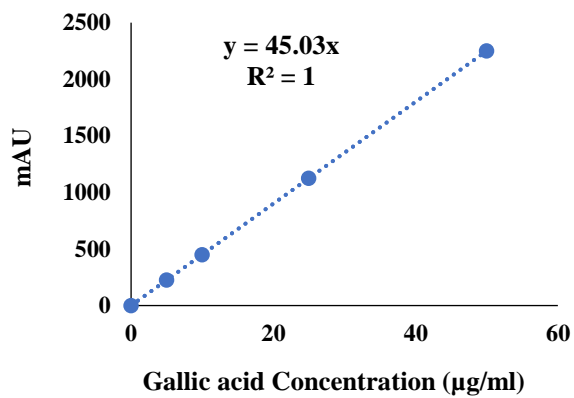

**S5 Fig.** Phenolic acids standard calibration curves

Supplement: S5 Fig — (PDF) [file pone.0321255.s005.pdf]

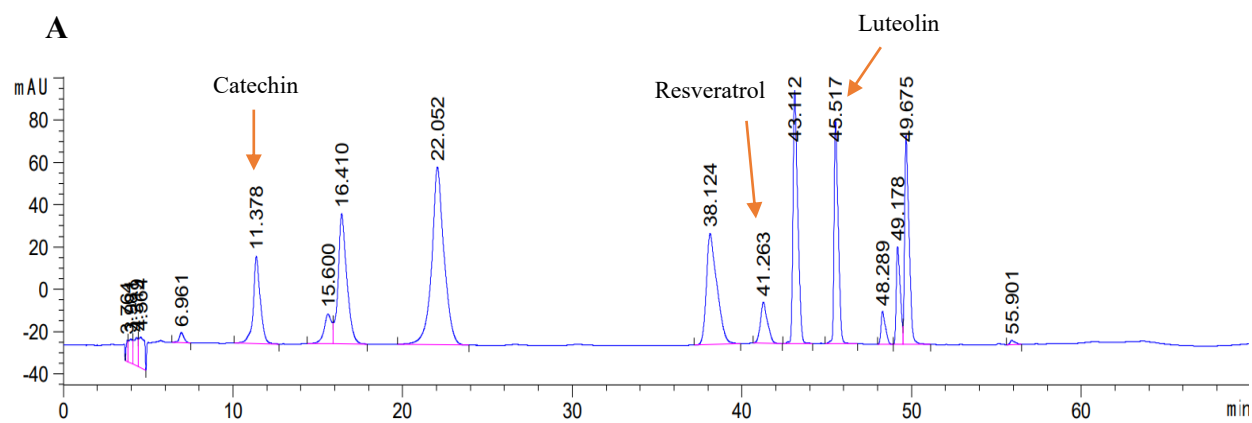

**S6 Fig.** Flavonoids HPLC chromatogram of reference

Supplement: S6 Fig — (PDF) [file pone.0321255.s006.pdf]

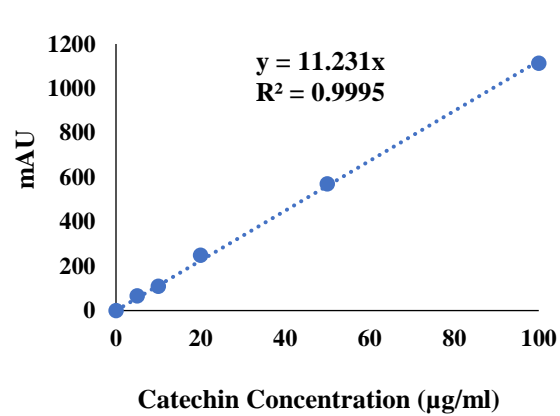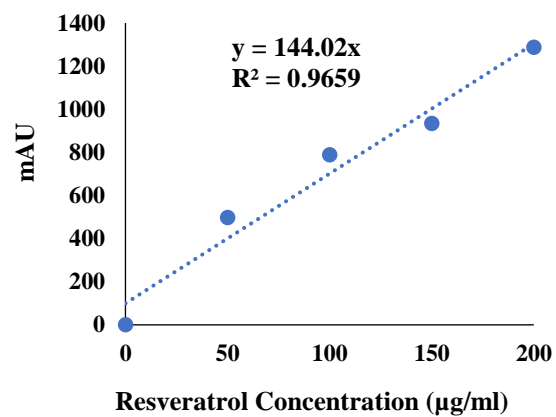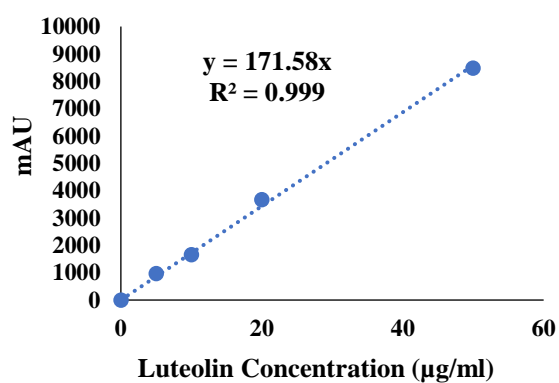

**S7 Fig.** flavonoids standard calibration curves

Supplement: S7 Fig — (PDF) [file pone.0321255.s007.pdf]

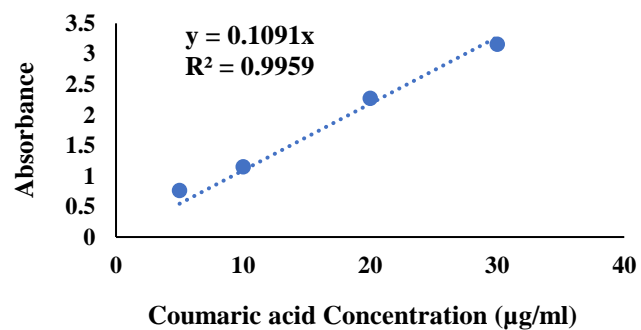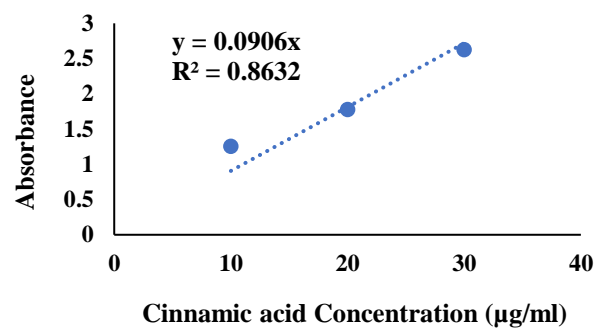

**S8 Fig.** PAL and TAL enzymes standard calibration curves

Supplement: S8 Fig — (PDF) [file pone.0321255.s008.pdf]

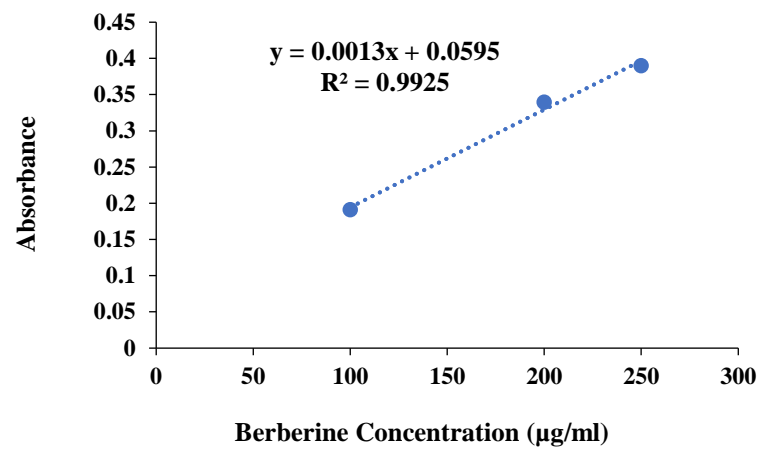

**S9 Fig.** Total alkaloid standard calibration curve

Supplement: S9 Fig — (PDF) [file pone.0321255.s009.pdf]

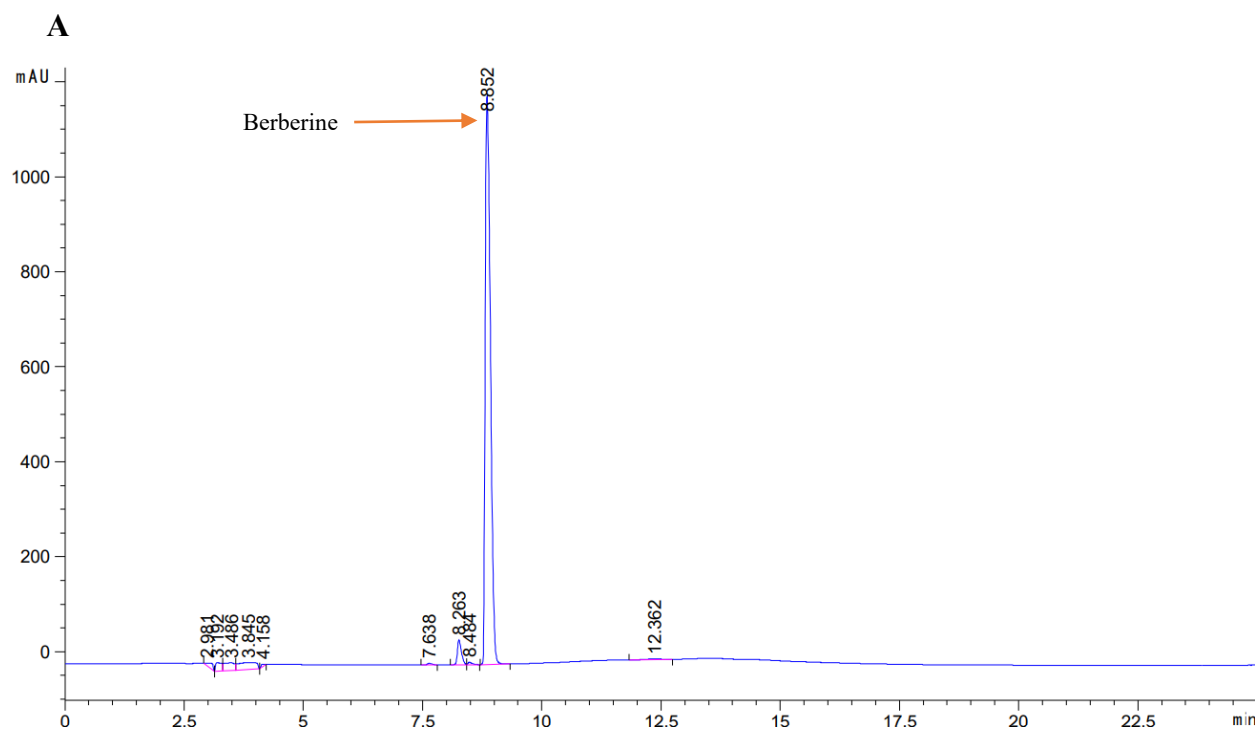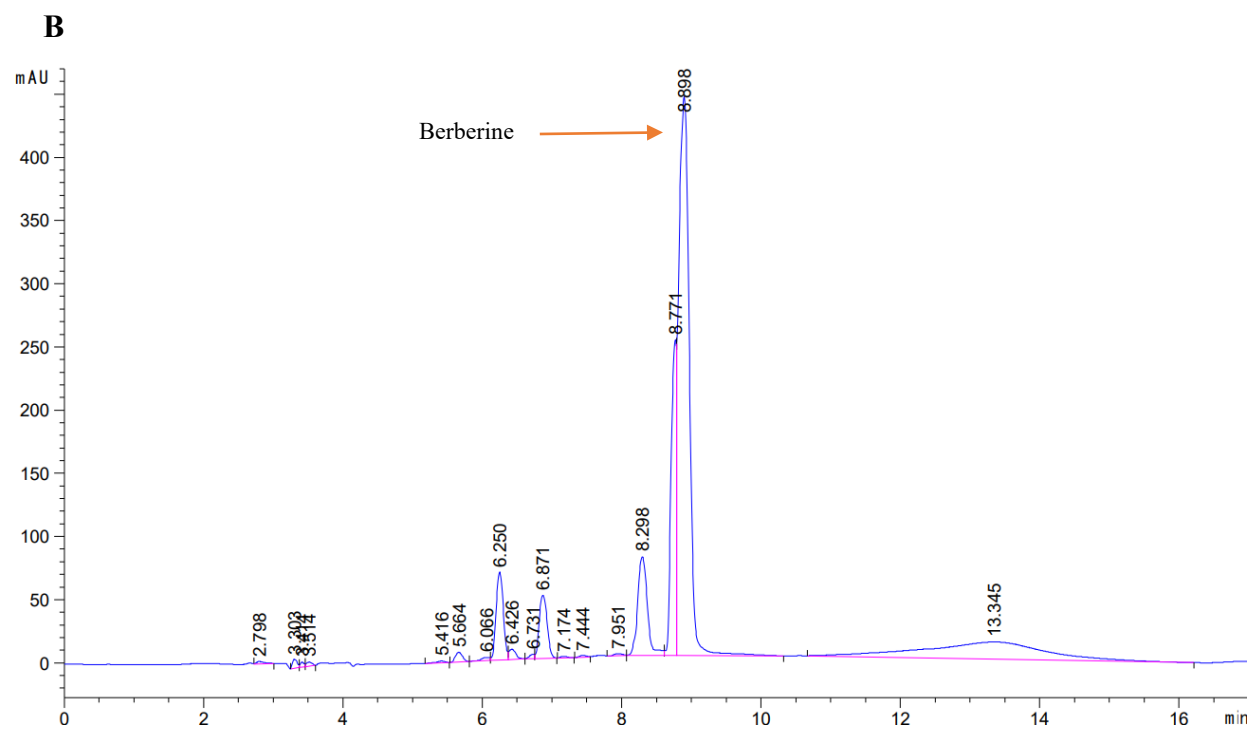

**S10 Fig.** Berberine HPLC chromatograms of (A) reference and (B) sample

Supplement: S10 Fig — (PDF) [file pone.0321255.s010.pdf]

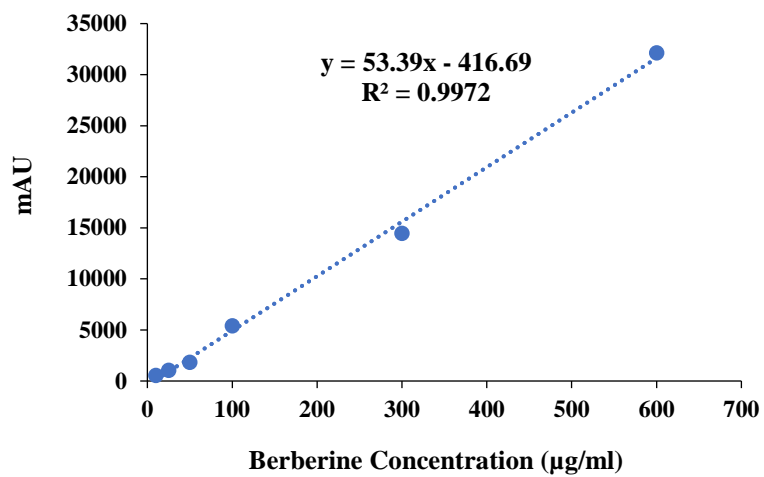

**S11 Fig.** Berberine standard calibration curve

Supplement: S11 Fig — (PDF) [file pone.0321255.s011.pdf]

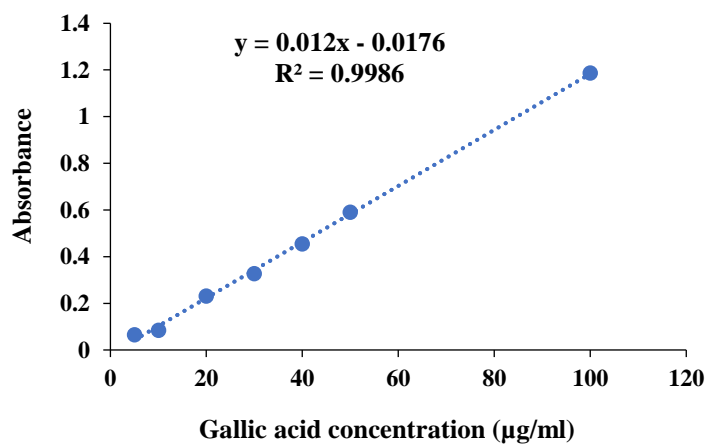

**S12 Fig.** FRAP standard calibration curve

Supplement: S12 Fig — (PDF) [file pone.0321255.s012.pdf]

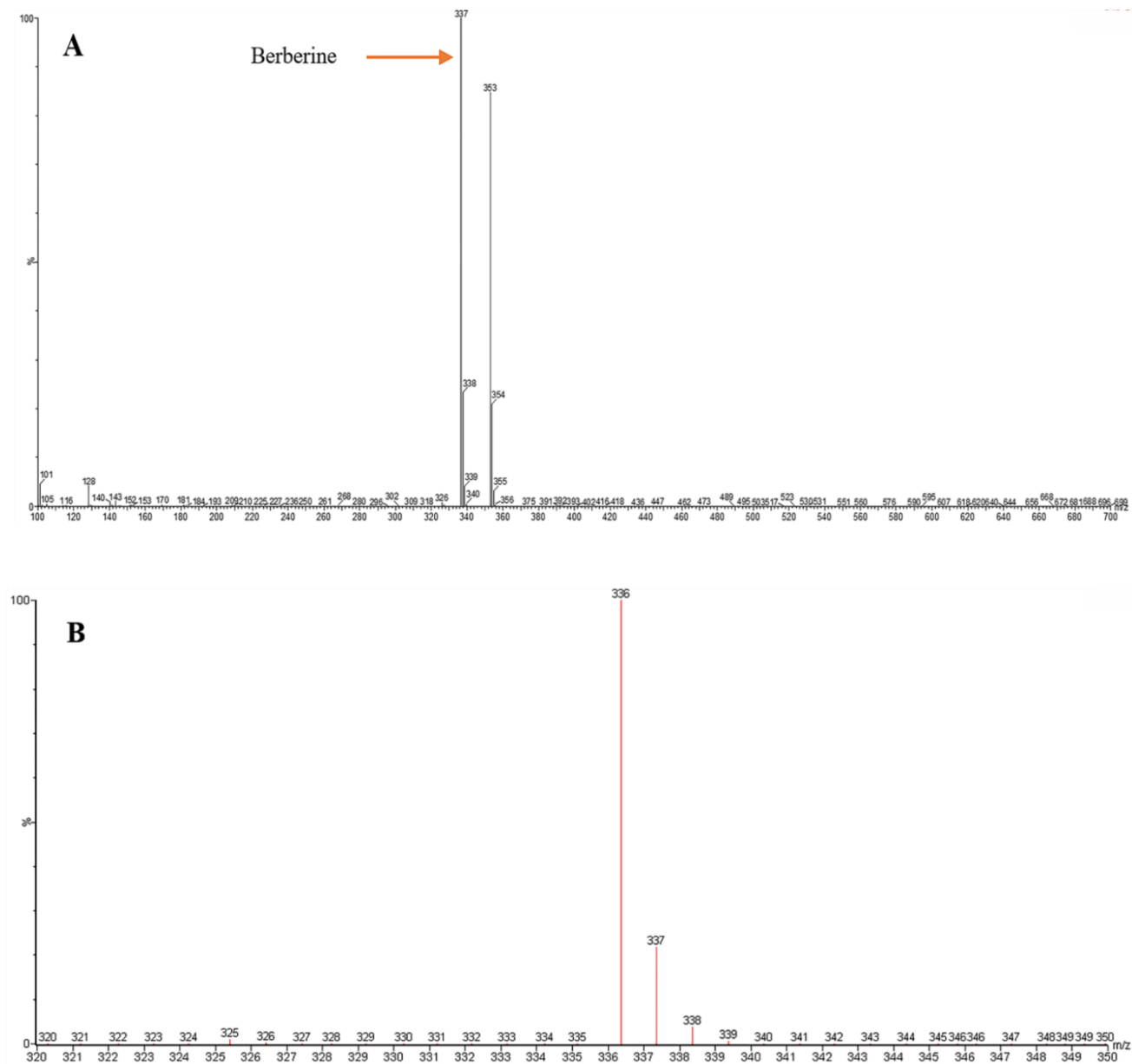

**S13 Fig.** Berberine mass spectrum

Supplement: S13 Fig — (PDF) [file pone.0321255.s013.pdf]
